# Exploring the Self-Assembly Capabilities of ABA-Type SBS, SIS, and Their Analogous Hydrogenated Copolymers onto Different Nanostructures Using Atomic Force Microscopy

**DOI:** 10.3390/ma11091529

**Published:** 2018-08-24

**Authors:** Nikolaos Politakos, Galder Kortaberria

**Affiliations:** 1 POLYMAT and Departamento de Química Aplicada, Facultad de Ciencias Químicas, University of the Basque Country UPV/EHU, Joxe Mari Korta Center, Avda. Tolosa 72, 20018 Donostia-San Sebastian, Spain; 2 “Materials + Technologies” Group, Chemical & Environmental Engineering Department, Basque Country University, Plaza Europa 1, 20018 Donostia-San Sebastian, Spain; galder.cortaberria@ehu.eus

**Keywords:** block copolymers, atomic force microscopy (AFM), polymer morphology, polymer nanostructures, self-assembly, polymer characterization, hydrogenated copolymers, polymer characterization

## Abstract

In this work, the self-assembled morphologies obtained for poly(styrene-b-butadiene-b-styrene) (SBS) and poly(styrene-b-isoprene-b-styrene) (SIS) ABA-type copolymers were investigated before and after hydrogenation of the polydiene block, which led to poly(styrene-b-ethylene)/poly(ethylene-b-styrene) (SEES) and poly(styrene-b-ethylene)/poly(propylene-b-styrene) (SEPS) copolymers, respectively. The evaluation of different morphologies was carried out using atomic force microscopy (AFM), analyzing the effect of various parameters such as the solvent and polymer concentrations employed for film casting (toluene, cyclohexane, or tetrahydrofurane with concentrations of 1 and 3 wt%), together with that of the annealing treatment (thermal annealing at room temperature, and 60, 80, and 100 °C). The effect of these parameters in combination with the chemical nature of the polydiene block led to different morphologies with different topographic aspects affecting the roughness (Ra) of the film.

## 1. Introduction

As several new applications emerge in the field of materials science, especially at the nanoscale level, new nanomaterials with improved properties are needed. Block copolymers are potentially suitable for this purpose due to their capability to self-assemble into ordered nanostructures. Currently, the development of new monomers and polymerization techniques can lead to various copolymers with interesting properties that can be controlled from their ordered nanostructures [1,2,3,4,5].

Immiscibility between covalently bonded blocks makes block copolymers form various nanostructures such as lamellae, spheres, cylinders, and double-gyroid [6,7,8], among others, in order to minimize their Gibbs free energy. The formation of well-ordered morphologies is mainly governed by the Flory–Huggins interaction parameter χ and the polymerization degree N (different χN values leading to morphologies that can be predicted through phase diagrams), together with other parameters such as the volume fraction of blocks and interfacial interactions [9,10]. Changes in N or χ parameters (block structure or nature) in block copolymers can alter obtained morphologies leading to new phase diagrams [11,12].

Block copolymers display many potential applications in nanotechnology [13,14,15], lubricants [1], drug delivery [16,17,18,19,20], nanopatterning [21,22,23,24,25], nanoporous membranes [26,27,28], biosensors [29,30,31,32,33], electronics [1,10], thin-film technology [34], semiconductors [35,36,37,38,39], construction [40,41], etc. As morphologies with highly ordered domains are needed for most of those applications, the different orientations of block copolymer domains in the self-assembled morphologies are very crucial and attract a lot of interest. Several methods are used for the ordering and alignment of block copolymers: chemical patterning [42,43], electric-field alignment [42,43,44], graphoepitaxy [42,43,44], contact line pinning [42], soft lithography [42], shear alignment [42,43], directional crystallization [42,43], and thermal or solvent vapor annealing [44], among others.

The casting of block-copolymer thin films from solutions onto different substrates is one of the ways of obtaining oriented nanostructures. This procedure involves the study and control of many parameters in order to obtain the desired nanodomains. The nature of the substrate, the polymer deposition method, solvent selection, and solvent removal method will determine the obtained morphology [45]. The choice of solvent and its selectivity toward one of the blocks is of crucial importance. Neutral solvents swell all blocks, while a solvent selective for one of the blocks will make the chains of this block swell and the chains of the other blocks collapse. The solvent evaporation rate can also have a strong effect on the generated nanostructures [34,42,46], as well as the thin-film thickness and the interactions between blocks and those at air and substrate interfaces [34,44], which also play a significant role in the final morphology. In the literature, the evolution of morphology was studied for different polymeric systems analyzing the effect of some of the abovementioned specific parameters, especially for copolymers containing polybutadiene (PB) [47,48,49] or poly(isoprene) (PI) [50].

As mentioned above, the affinity of different solvents for copolymer blocks can lead to different nanostructures and organization. Through thermodynamics, the affinity of one of the blocks for the solvent is the driving force for the formation of ordered nanostructures. The evaporation rate of the solvent for a drop-casted block-copolymer thin film can also determine the nanostructure, as, depending on the solubility of each block, one of the blocks can rearrange and move toward the surface, relative to the other block. Thermal annealing is also very important, as it can provide mobility to the polymeric chains for their ordering.

The solubility, and subsequently, the affinity of a solvent for a polymer involves three important parameters. These are dispersion contribution (δ_d_), the dipole contribution (δ_p_), and the hydrogen-bond contribution (δ_h_) to the solubility parameter. These parameters, combined with Equation (1),

δ^2^ = δ_d_^2^ + δ_p_^2^ + δ_h_^2^, (1)

can give the value of the solubility parameter, according to Hansen [51]. By combining Equation (1) with Equation (2) [52],

χ_sp_ = (aV/RT) × ((δ_ds_ − δ_dp_)^2^ + 0.25(δ_ps_ − δ_pp_)^2^ + 0.25(δ_hs_ − δ_hp_)^2^), (2)

solubility among a polymer and a solvent can be calculated using the Hansen method [51]. By maintaining a constant temperature of 298 K (room temperature, RT), using the different solubility parameters and volume (V) for each solvent, the solvent–polymer interaction (χ_sp_) parameter can be calculated. The lower the χ_sp_ parameter, the higher the affinity of the polymer for the solvent [51,53].

Triblock copolymers of poly(styrene-b-butadiene-b-styrene) (SBS) and poly(styrene-b-isoprene-b-styrene) (SIS) are polymers that are widely used in various applications, such as in modified asphalt for studying the physical and rheological properties of asphalt during aging (SBS as the main component) [54], or to study their viscoelastic behavior (in the case of SIS) [55]. The PB or PI as one of the blocks has a double bond that is easy to modify, creating new polymeric blocks with new properties for new applications [11,12,56]. In addition, these triblock copolymers can form different nanostructures (cylindrical, cubic bicontinuous, and lamellae) depending on their molecular weight, the sequence of the blocks, and their volume fraction. It is clear that these copolymers are good candidates for use in construction or nanotechnology due to their self-assembly [57,58,59].

The aim of this manuscript was to investigate the effect of sample preparation (polymer solution concentration, solvent nature, and annealing treatment) on the morphologies and surface topography of specific poly(styrene-b-isoprene-b-styrene) (SIS) and poly(styrene-b-butadiene-b-styrene) (SBS) ABA-type copolymers and their hydrogenated poly(styrene-b-ethylene)/poly(propylene-b-styrene) (SEPS) and poly(styrene-b-ethylene)/poly(ethylene-b-styrene) (SEES) analogs. Thin films of all copolymers were casted from their toluene, cyclohexane, and tetrahydrofuran solutions with concentrations of 1 or 3 wt% onto glass wafers. Samples were annealed at different temperatures (room temperature, and 60, 80 and 100 °C). Different morphologies were obtained depending on the solvent, concentration, and annealing temperature, revealing the importance of the control of such parameters on the formation of desired nanostructured domains. These copolymers can show interesting nanostructures that can be controlled by changing different parameters. They can be used in nanopattering, as membranes or as points where molecules/particles can be introduced or trapped. It is very important to notice that the use of these triblock copolymers with polydiene as one of the blocks can lead to polymeric materials with different properties such as thermal, viscoelastic, and degradation stability. Finally, the easy modification of these commercial copolymers (due to the double bond of the polydiene) can lead to new materials that can be adjusted to the need of the market.

## 2. Materials and Methods

### 2.1. Materials

SIS and SBS copolymers were used as received. The SIS triblock copolymer was obtained from Kraton Polymers with a total molecular weight of 58,200 g mol^−1^ (M_W,PS_ = 17,460 g mol^−1^ and M_W,PI_ = 40,740 g mol^−1^) and a weight fraction, Φ_ps_ = 0.3. The SBS triblock copolymer was kindly supplied by Repsol-YPF (Dynasol C540, Madrid, Spain) with a total molecular weight of 75,000 g mol^−1^ (M_W,PS_ = 30,000 g mol^−1^ and M_W,PI_ = 45,000 g mol^−1^) and a weight fraction, Φ_ps_ = 0.4. The hydrogenation procedure for obtaining SEPS and SEES was conducted with *p*-toluenesulfonyl hydrazide (Sigma-Aldrich (Saint Louis, MO, USA), 97%) and it is described elsewhere [60]. Toluene (Lab Scan, HPLC, 99.8%, Samut Sakhon, Thailand), cyclohexane (Panreac, 99.5%, Barcelona, Spain), and tetrahydrofuran (THF; Lab-scan, 99.8%, Samut Sakhon, Thailand) were used as solvents without any further purification.

### 2.2. Thin-Film Preparation

Copolymer solutions (1 and 3 wt%) in toluene, THF, and cyclohexane were left stirring for 24 h at room temperature. Then, solutions were drop-casted onto glass wafers for 24 h in order to ensure the full evaporation of the solvent. Films were then annealed in a high-vacuum oven (Binder, Tuttlingen, Germany) for 24 h at different temperatures: room temperature, and 60, 80 or 100 °C.

### 2.3. Morphological Characterization

The surface morphology of the thin films was characterized using atomic force microscopy (Dimension 3100/Nanoscope IVA, Veeco, New York, NY, USA) (from Digital Instruments Santa Barbara, CA, USA). The tapping mode in air was employed using an integrated silicon tip/cantilever (125 μm in length) at a scan rate of 1.0 Hz and a resonant frequency of ~300 kHz [61,62,63].

## 3. Results

The change of solvent can lead to different affinities for the blocks, and this can lead to different ordered nanostructures. It is very important to know the solubility parameters between the polymeric blocks and the solvents, so as to explain some nanostructures. It must be pointed out that, for copolymers used in this work, the middle block was a polydiene (PB or PI), and following hydrogenation, a polyolefin (polyethylene (PE) or PE/polypropylene (PP)); as such, their glass transition temperatures were lower than room temperature.

In Table 1, all solubility parameters for dispersion, dipole, and hydrogen bonding for all solvents and all polymeric blocks used in this work are shown, as calculated using Equations (1) and (2) or as found in the literature [51,64]. On the other hand, Table 2 shows the χ_sp_ parameters for the polymer/solvent systems based on PS, PB, PI, PE, and PEP polymers, and toluene, cyclohexane, and THF solvents, as calculated using Equation (2) [52]. Based on Equations (1) and (2), the solubility of the polymers in the solvents could be evaluated [65,66].

From Table 1 and Table 2, an estimation concerning the affinity of each polymeric block for solvents could be done. Even though the middle block was different in all copolymers (PB, PI, PEP, and PE), their chemical structure and behavior were very close. It was assumed that similar solvents would present similar solubility with polydienes (PB and PI) and their hydrogenated analogs (PEP and PE). As shown in Table 2, in the case of the PS block, THF could be considered as the most selective, followed by toluene and cyclohexane. For PI and its hydrogenated PE and PEP analogs, the same behavior was observed with all solvents. Toluene presented the highest selectivity, followed by cyclohexane, both favoring selectivity for the middle block. In the case of PB, the highest selectivity was observed for toluene, followed by THF and cyclohexane. Thus, a different behavior can be expected for the copolymer with a PB middle block as compared to the other ABA copolymers, due to its different solvent affinity, as seen in Table 2. It is worth noting that, for all copolymers, THF was more selective for PS than for the middle block, while toluene and cyclohexane were more selective for the middle block than for PS. For the PS-b-PEP-b-PS/toluene copolymer/solvent system the selectivity was very similar, indicating that this solvent could be neutral for both blocks, with slight selectivity for PEP. For the PS-b-PB-b-PS/cyclohexane system, on the other hand, the solvent was more selective for the PB central block.

**Figure 1:**
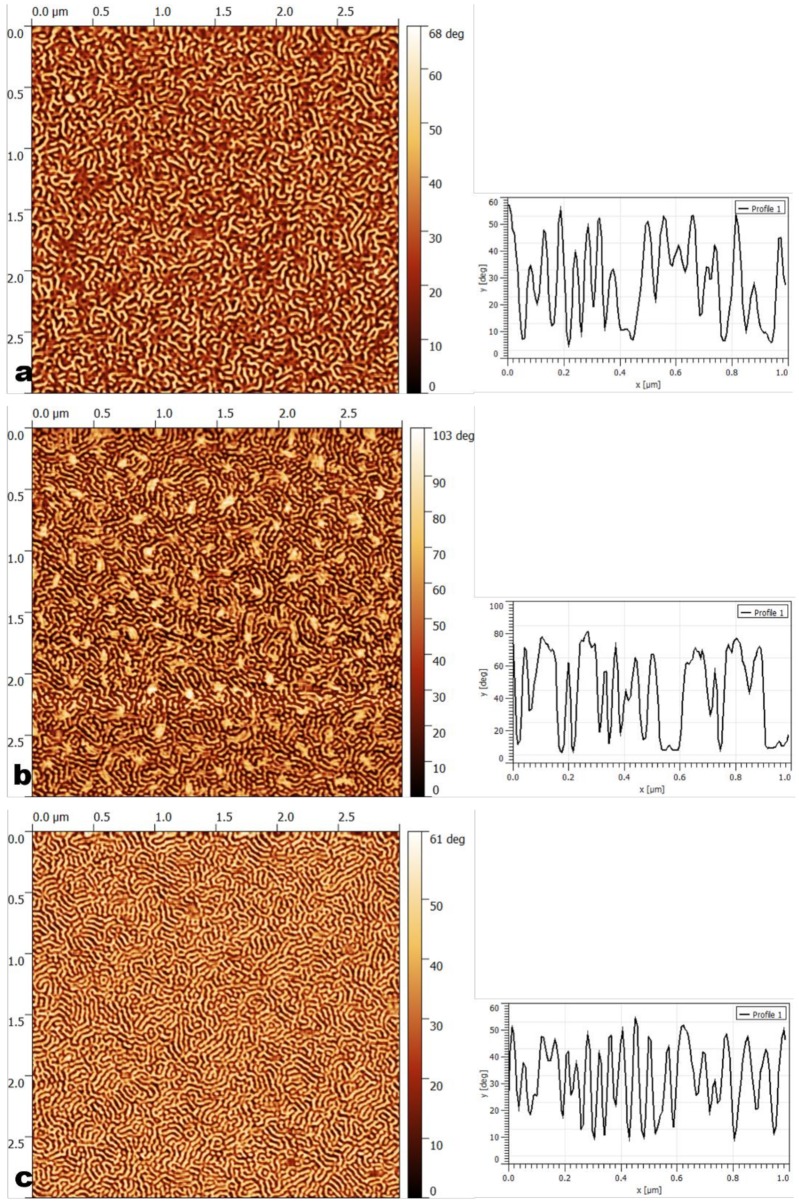
Atomic force microscopy (AFM) phase images (3 μm × 3 μm) and corresponding cross-sections (1 μm × 1 μm) of worm-like type morphologies: (**a**) worm-like (3 wt% poly(styrene-b-ethylene)/poly(propylene-b-styrene) (SEPS) casted from toluene annealed at 100 °C); (**b**) worm-like with crystals (1 wt% SEPS casted from toluene annealed at 60 °C); (**c**) worm-like with perpendicular cylinders (1 wt% SEPS casted from toluene annealed at room temperature (RT)).

**Figure 2:**
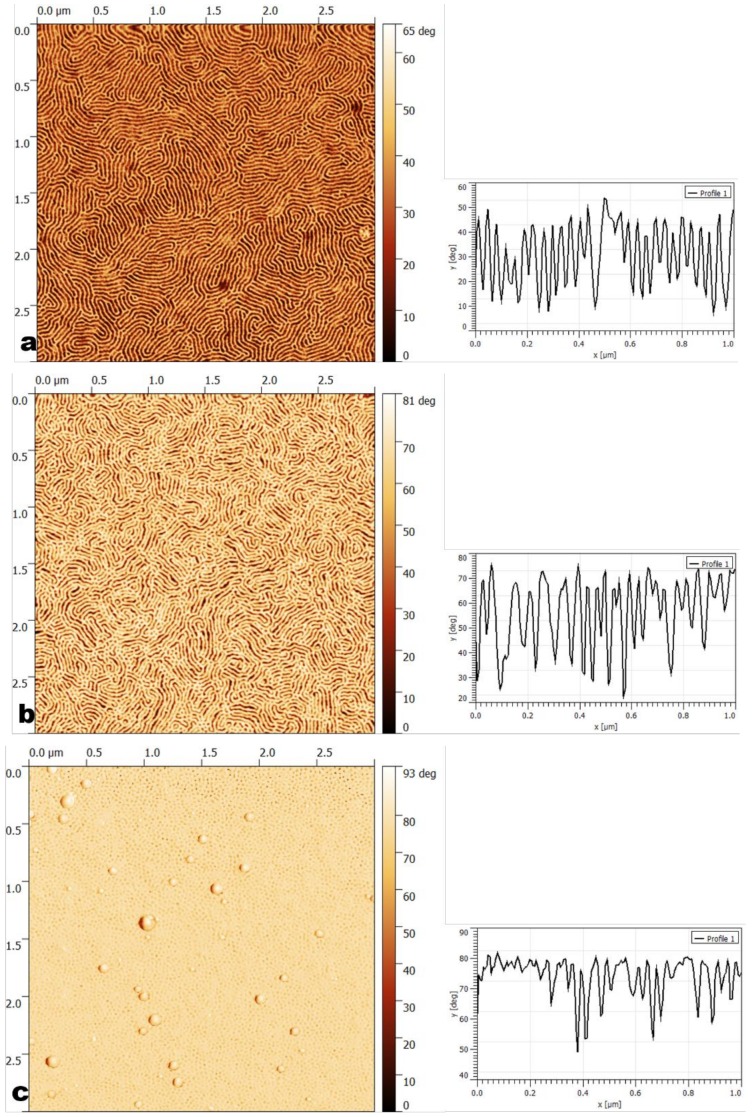

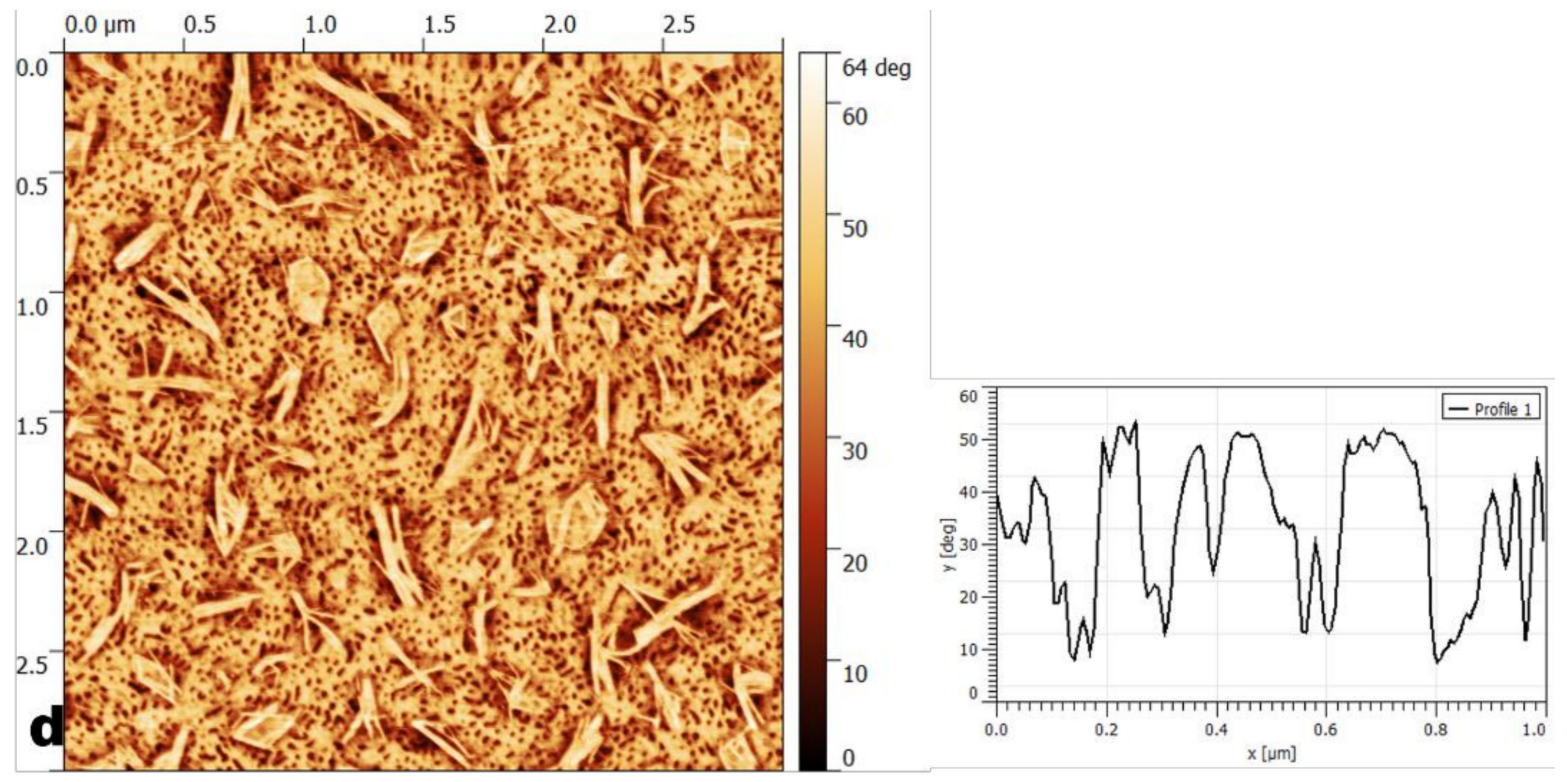
AFM phase images (3 μm × 3 μm) and the corresponding cross-sections (1 μm × 1 μm) of cylindrical morphologies: (**a**) parallel cylinders (3 wt% poly(styrene-b-isoprene-b-styrene) (SIS) casted from toluene annealed at RT); (**b**) parallel and perpendicular cylinders (3 wt% SIS casted from tetrahydrofuran (THF), annealed at RT); (**c**) perpendicular disordered cylinders (3 wt% poly(styrene-b-butadiene-b-styrene) (SBS) casted from toluene, annealed at RT); (**d**) perpendicular cylinders with large crystal-like domains.

Once the selectivity of the solvents for the blocks was established, ordering occurred depending on various parameters such as the annealing temperature, and the concentrations of the copolymer solution and solvent. The effects of those parameters on the obtained morphologies were subsequently analyzed. The obtained structures are gathered in Table 3, while in Table 4 a nomenclature of the parameters used for all samples can be seen. The AFM images are shown in Figure 1 and Figure 2. Seven main morphologies could be observed: worm-like, worm-like with perpendicular cylinders, worm-like with crystalline domains, parallel cylinders, a mixture of parallel and perpendicular cylinders, disordered perpendicular cylinders, and perpendicular cylinders together with large crystal-like domains. Figure 1 shows worm-like nanostructures: worm-like (a), worm-like with crystalline domains (b), worm-like with perpendicular cylinders (c). Figure 2 shows cylinder-based nanostructures: parallel cylinders (a), mixture of parallel and perpendicular cylinders (b), disordered cylinders (c), and perpendicular cylinders with large crystal-like domains (d).

It is clear that the morphologies were greatly affected by the chemical nature of the copolymers and adjustable parameters such as the solvent or annealing treatment. During solvent evaporation, blocks with affinity for the solvent tend to orient themselves toward the surface. Annealing treatment can also help some blocks move toward the surface or to better organize themselves due to the increased mobility. Generally, the middle block (polydiene and polyolefine) presented a higher affinity for toluene and cyclohexane, while the end block (PS) had a higher affinity for THF. When toluene and cyclohexane solutions were casted onto glass wafers, the middle block moved toward the outermost surface due to its affinity for the solvent and the surface tension. Copolymer domains with lower surface tension (PI and PB) present this behavior in order to minimize surface energy [67,68,69]. In the case of block copolymers, the nature of the substrate and the affinity that each block has for the substrate and/or air is very crucial. The surface tension to air and the interfacial tension with the substrate can lead to different affinities, and hence, different morphologies. Surface tension has a strong influence on the morphology of polymer thin films. The chemical modification of the substrate can change the morphology of the same block copolymer. Polymeric thin films form microdomains that are controlled by preferential interactions between blocks and the substrate. In terms of multilayers, films can be symmetric (the same block segregates to both interfaces upon annealing) or asymmetric (each interface adsorbs a different component) [70,71,72,73]. For samples prepared with THF, due to its higher affinity for the PS block, it moved toward the surface. In this case, the higher evaporation rate of the THF solvent was crucial and affected the final morphologies. Finally, the concentration of casted solutions also plays a significant role in the final morphologies, since it determines film thickness [42,74,75,76,77]. All these parameters are further analyzed in the upcoming sections.

### 3.1. Effect of Block Nature and Solution Concentration

In the case of the SEPS copolymer, highly ordered worm-like morphologies were observed, especially for thinner films obtained from 1 wt% solutions. Increasing the film thickness (3 wt% solution), a worm-like morphology was obtained, but with the presence of cylinders, oriented perpendicularly to the surface. The SIS copolymer films prepared using 3 wt% solutions showed some perpendicular cylinders together with crystal-like large domains, cylinders with a parallel orientation, or cylinders with both perpendicular and parallel orientations. For thinner films (1 wt% solutions), worm-like morphologies with some cylinders with different ordering and orientation were obtained. For SEES, a worm-like ordered morphology was observed only for samples annealed at 100 °C (for both 1 and 3 wt% solutions). For SBS, a mixture of morphologies was observed: worm-like (also with perpendicular cylinders for 3 wt% solutions), parallel cylinders (1 wt%), and perpendicular disordered cylinders (1 and 3 wt%). As a general observation for systems with polydiene as the middle block, SIS and SBS generally presents both types of cylinder morphologies (perpendicular or parallel to the substrate and/or a mixture of both) [78]. In our case, SIS presented parallel cylinders oriented toward the surface (for 1 wt% cyclohexane at RT, 3 wt% cyclohexane at RT, and 3 wt% toluene at RT) and a mixture of parallel/perpendicular cylinders (for 1% toluene at RT and 3 wt% THF at RT). In addition, SBS presented a more worm-like morphology and a mixture of ordered nanostructures: worm-like/perpendicular cylinders for 3 wt% toluene at 60 °C, parallel cylinders for 3 wt% cyclohexane at RT, and disordered perpendicular cylinders for 1 wt% and 3 wt% toluene at RT. Finally, it is worth noting that SIS was the only one showing a mixed morphology of perpendicular cylinders with crystal-like domains. These morphologies were found for the 3 wt% solutions of all three solvents and an annealing treatment at 60 °C. The reason for the appearance of these crystal-like domains could be the fact that the SIS copolymer presented a mixture of PI microstructures (*cis*/*trans* 1,4-, 3,4-, and 1,2-) and a small percentage of an SIS copolymer.

### 3.2. Effect of Annealing Temperature

Starting with samples annealed at RT, SEPS showed a worm-like morphology with perpendicular cylinders (3 wt%) and a mixture of parallel/perpendicular cylinders (1 wt%). Its precursor (SIS) showed parallel cylinders at 3 wt% and a mixture of worm-like morphology, parallel cylinders, and parallel/perpendicular cylinders at 1 wt%. SEES showed a disordered morphology, while its SBS precursor showed three different morphologies. For samples prepared with 1 wt% solutions, a disordered morphology was found for THF, a worm-like morphology was found for cyclohexane, and a worm-like morphology with perpendicular cylinders was found for toluene. For samples with 3 wt% solutions, a disordered morphology was obtained for THF, while parallel cylinders were obtained for cyclohexane and perpendicular cylinders were obtained for toluene.

Regarding the samples annealed at 60 °C, SEPS showed some crystalline PEP domains combined with a worm-like morphology for 1 wt% solutions. For samples with 3 wt% solutions, the same morphology as that seen at RT was obtained (worm-like with perpendicular cylinders). For SIS (compared to morphologies obtained at RT), the ordering was faint, showing disordered and worm-like morphologies and perpendicular cylinders with the presence of crystal-like domains at 1 wt%, and perpendicular cylinders with crystal-like domains at 3 wt%. SEES and SBS showed disordered morphologies.

For samples annealed at 80 °C, SIS SEES, and SBS showed generally disordered structures. SEPS showed a worm-like morphology at 1 wt%, while, at 3 wt%, a worm-like morphology was generated for THF, a worm-like morphology with perpendicular cylinders was generated for cyclohexane, and perpendicular/parallel cylinders were generated for toluene. The influence of solvent evaporation rate was clearly shown in the final ordering. Solvents such as THF which evaporate quickly do not give an appropriate time for relaxation of the polymeric chains, so as to achieve thermodynamically stable nanostructures.

Finally, in the case of the samples annealed at 100 °C, SEPS showed only a worm-like morphology, probably due to the migration of PS to the outermost surface due to the energy obtained from the thermal treatment. SEES showed some nanostructures, such as a worm-like morphology at 1 and 3 wt% solutions in THF and cyclohexane. For the remainder, disordered structures were observed, probably due to the increased mobility of the PS chains.

### 3.3. Effect of Solvent Nature

In the case of cyclohexane, a worm-like morphology only or that with perpendicular cylinders was observed for SEPS at 1 and 3 wt% solutions, respectively. The remainder of the samples showed disordered morphologies with the same solvent. Similar results were found for toluene, whereby SEPS showed a worm-like morphology with crystals at 1 wt%, and a worm-like morphology with perpendicular cylinders at 3 wt%. The remainder of the samples showed disordered morphologies. Finally, for THF, SEPS adopted a worm-like morphology at both 1 and 3 wt% solutions, while SIS and SEES (3 wt%) showed perpendicular cylinders and a worm-like morphology, respectively.

Generally, a worm-like morphology was favored for THF, while a cylindrical one was favored for toluene and cyclohexane. THF favored a worm-like morphology and perpendicular cylinders with large domains, while toluene favored a worm-like morphology with crystals, disordered perpendicular cylinders, and parallel/perpendicular cylinders. Finally, cyclohexane favored a worm-like morphology with perpendicular cylinders and perpendicular cylinders with large domains. In the case of THF, with the highest evaporation rate in combination with chain mobility after annealing, blocks did not have enough time for the generation of ordered morphologies, obtaining a worm-like morphology or a mixture of morphologies. Even if worm-like morphologies were observed for all solvents, the majority (12) were obtained for THF, and 10 for cyclohexane and toluene (especially for SEPS, SIS, and SEES). The opposite effect could be observed for the ordered morphologies (parallel or perpendicular cylinders, or a mixture). Cyclohexane presented eight cases (for SEPS, SIS, and SBS), and toluene presented seven cases (for SEPS, SIS, and SBS), while only three cases were obtained with THF (for SIS).

Some general considerations could be derived after analyzing the effect of different parameters, where the highest order was shown in the SEPS morphologies. Temperature also played a significant role, obtaining the highest ordered morphologies at RT. For samples with a PEP middle block, crystals were formed at 60 °C and at 80 °C. At higher temperatures, the mobility of the PS end blocks, due to the thermal energy, led to worm-like ordered domains. Even though the middle block was generally present at the outermost surface (for cyclohexane and toluene), the temperature made it possible for PS chains to migrate to the outermost surface, leading to worm-like morphologies. For samples casted from cyclohexane and toluene, the middle block was expected to be at the outermost surface mixed with PS for higher annealing temperatures, due to its increased mobility. For both solvents, SEPS showed quite a similar morphology, with differences in ordering, orientation, and the presence or absence of crystals. The affinity between blocks and solvent also played an important role. For toluene, the affinity for PS and PEP was similar (0.170 and 0.120, respectively), while, for cyclohexane, the affinity for PS was 0.360, and, for PEP, it was 0.160. Thus, the evaporation rate of cyclohexane from the middle block was slower when compared to that of PS. Therefore, the middle block had more chance to relax and assume a stretch configuration, while the PS blocks tended to aggregate so as to minimize contact [47,48]. This can lead to a surface with a majority of middle-block domains. The evaporation rate also plays an important role in crystal nucleation [46,79,80]. As the evaporation rate of toluene is lower than that of cyclohexane, the nucleation mechanism is favored. For samples casted from toluene, the ordering was due to the slow evaporation rate, while, for cyclohexane, it was due to the different affinity of the blocks for the solvent. On the contrary, for THF, the evaporation rate was higher, and PS did not have time to migrate to the outermost surface. Samples casted from THF showed a worm-like morphology, and with the increasing annealing temperature, the PS block had enough energy to organize, showing a more ordered worm-like morphology. Regarding the effect of thickness (related to solution concentration), it is worth noting that thicker films showed more ordered nanostructures (25, where 10 of them had a cylindrical morphology) than the thinner ones (21, where only 5 of them had a cylindrical morphology).

### 3.4. Detailed Analysis of the Effect of Parameters on Morphology and Topography

**Figure 3:**
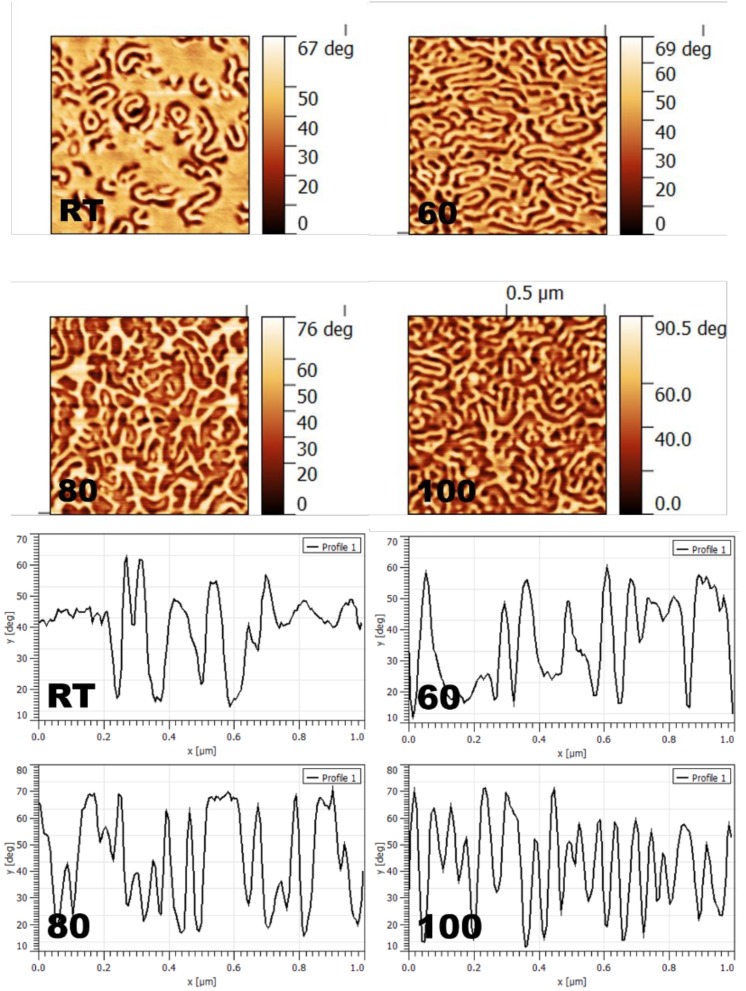
AFM phase images and corresponding cross-sections (1 μm × 1 μm) showing the evolution of the worm-like morphology with annealing temperature for the SEPS sample casted from a 3 wt% solution in THF.

**Figure 4:**
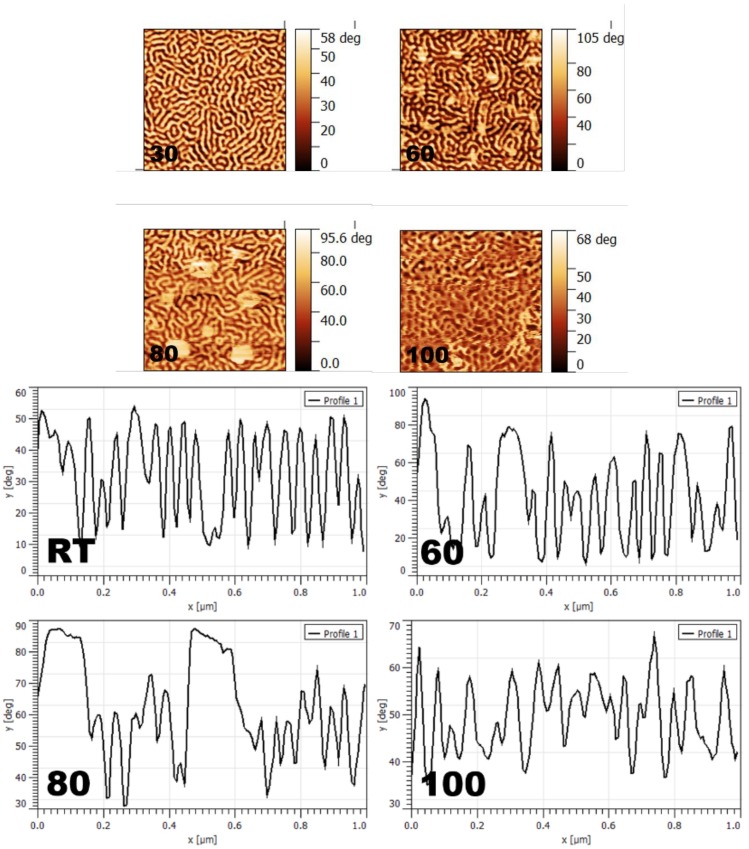
AFM phase images and corresponding cross-sections (1 μm × 1 μm) showing the evolution of self-assembly and crystallization with annealing temperature for the SEPS sample casted from a 1 wt% solution in toluene.

In order to analyze, in more detail, the effect of temperature, solvent, concentration, and nature of blocks on sample morphology and topography, a series of identical samples prepared under different conditions were evaluated. Starting with the effect of solvent and annealing temperature, two series obtained from an SEPS sample with THF and toluene are presented at different temperatures in Figure 3 and Figure 4. As the first example, Figure 4 shows the evolution of the self-assembly and ordering of the SEPS sample casted from 3 wt% solutions of THF at different annealing temperatures, in order to analyze the effect of annealing treatment. The evolution of the worm-like morphology with temperature can be observed. THF presented a higher affinity for PS blocks than for PP/PE ones; however, the worm-like morphology was not fully created at RT due to the fast evaporation rate of THF, which did not give enough time for the structure to order [79]. As solvents with higher affinity for one block generally give extra volume to that block [49], it can be assumed that PS gained this extra volume. Therefore, at RT, the outermost surface could be a mixture of both blocks. Increasing the annealing temperature, the mobility of PS blocks increased due to thermal energy, maximizing the probability of thermodynamic equilibrium [46]. Regarding topography, the presence of PS at the outermost surface seems to be clear, as roughness increased with temperature (from 9.27 to 11.70, and from 14.04 to 15.27).

Regarding SEPS samples casted from toluene, Figure 4 shows the evolution of morphologies with annealing temperature, with the appearance of crystalline domains together with the self-assembly process.

The evolution of a worm-like morphology can be seen, together with the appearance of crystals 100 nm in size and in a polygonal form at 60 °C, which grew at 80 °C (125 nm). Due to the lower evaporation rate of toluene, samples had a longer time for ordering [79]; thus, even at RT, the worm-like morphology was formed, unlike samples casted from THF (higher evaporation rate), in which a worm-like morphology was not obtained at RT (see Figure 3). The outermost surface was mostly PP/PE (due to a slightly better affinity than PS). When the annealing temperature was increased to 100 °C, a lower degree of ordering could be found, probably due to the increased mobility of PS blocks that could migrate toward the surface, obtaining a morphology composed of overlapping chains of middle and end blocks. PP/PE crystals observed at 60 and 80 °C, did not appear for samples annealed at 100 °C. The roughness increased upon increasing the annealing temperature, especially due to the existence of larger crystals, before decreasing again for samples annealed at 100 °C, without crystal formation (roughness values from RT to 100 °C: 11.06, 19.36, 13.15, and 7.50). The formation of tiny spherical or clumpy crystallites can be ascribed to the confined crystallization in hard block-rich domains [81]. When both the crystalline and amorphous blocks are strongly segregated in the molten state and the degree of crystallinity is relatively low, this type of crystallinity behavior can be expected. At temperatures of 50 °C and below, crystals are fully confined by the pre-existing cylinder microdomains; however, at temperatures higher than 50 °C, the crystals generally grow parallel to the axes of the cylindrical microdomains. Moreover, it can be seen that the nucleation density quickly increased with decreasing temperature. At low supercooling (high temperatures), such as 100 °C, very few nucleation sites were found, as seen in Figure 4. However, at larger supercooling (low temperatures), such as 60 and 80 °C, there was a significant increase in the density of nucleation sites [82].

Moreover, a detailed analysis of the film roughness for all samples is presented in Figure S1 (Supplementary Materials). In Figure S1, it can be observed how the roughness changed based on solvent (Figure S1a) and solution concentration (Figure S1b) for SBS, SEES, SIS, and SEPS.

**Figure 5:**
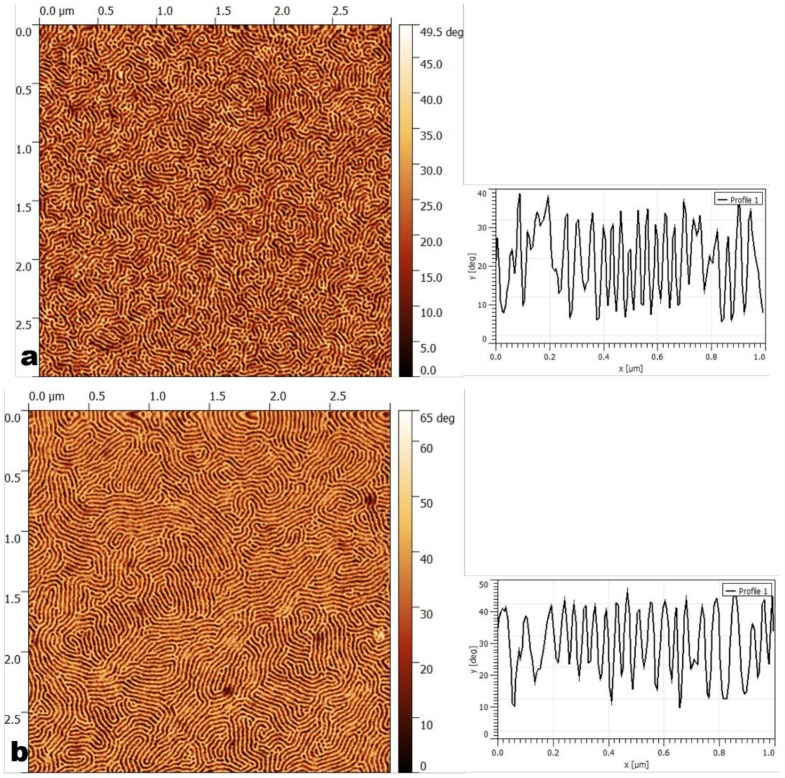

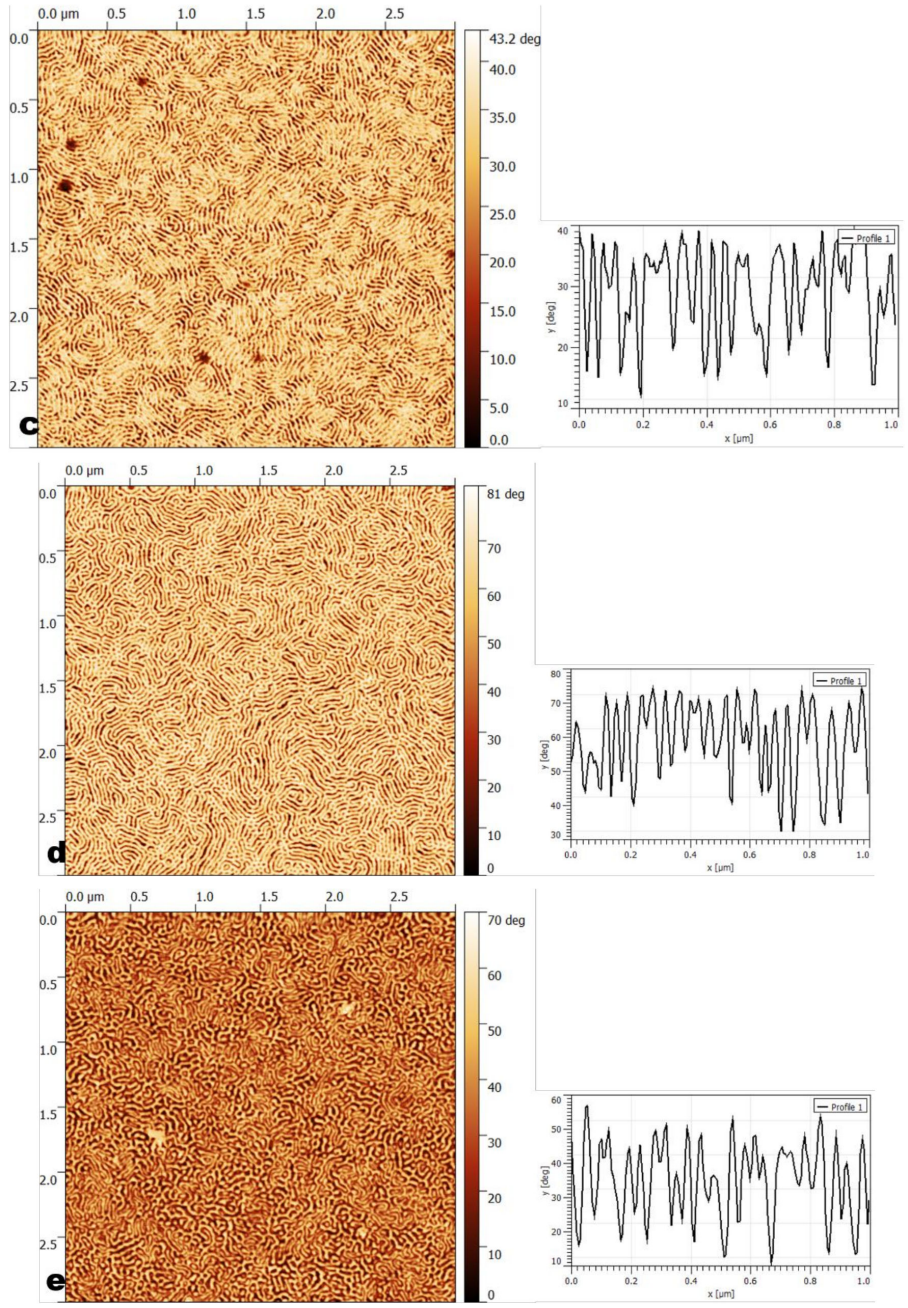

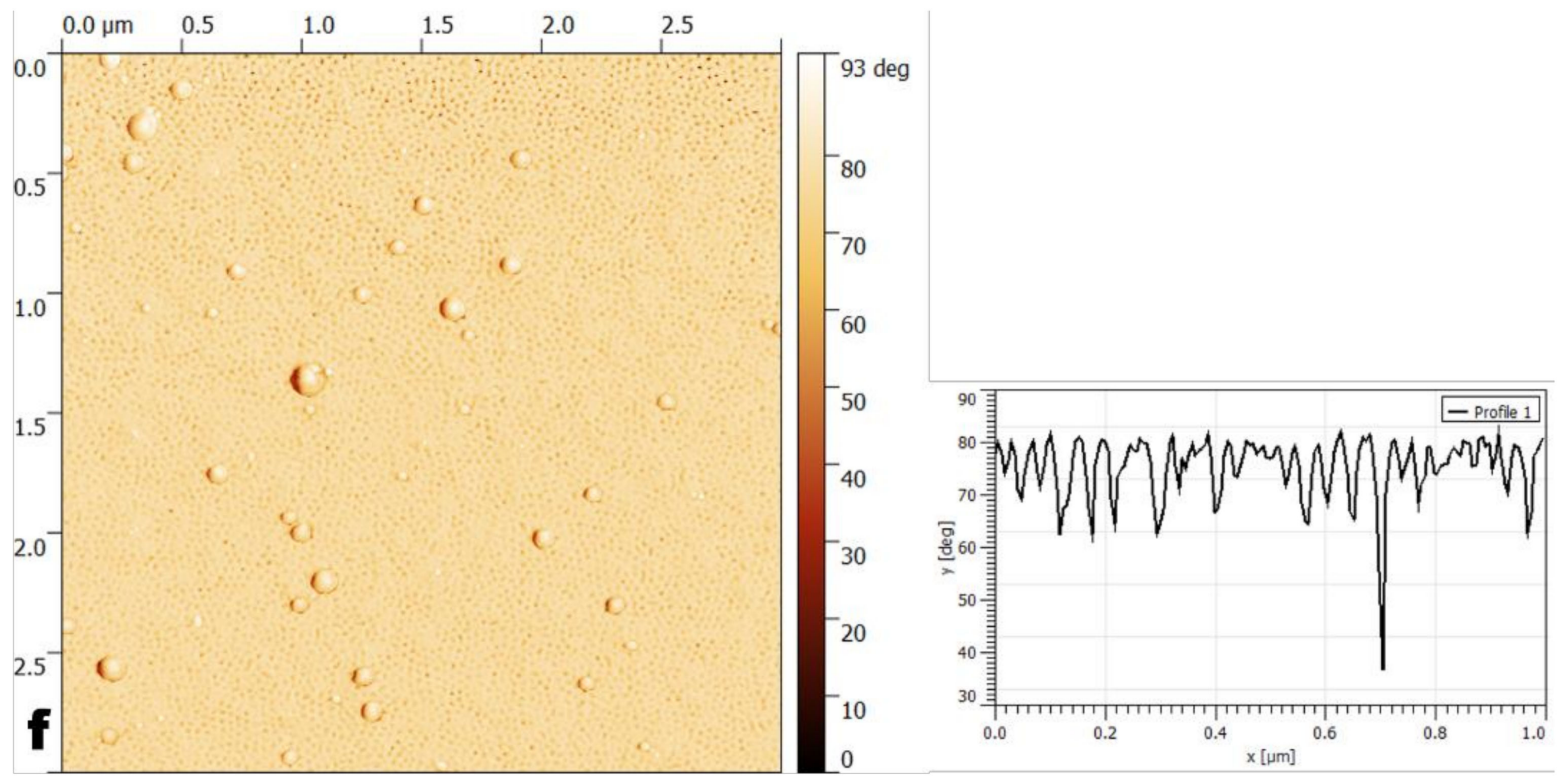
AFM images (3 μm × 3 μm) and corresponding cross-sections obtained for different copolymer films prepared under different conditions: (**a**,**b**) SIS casted from 1 and 3 wt% toluene solutions, respectively, at RT; (**c**,**d**) SIS casted from 3 wt% solutions in cyclohexane and THF, respectively, at RT; (**e**,**f**) SEPS and SBS, respectively, casted from 3 wt% solutions in toluene, at RT.

In order to obtain some basic conclusions concerning the effect of solution concentration, the nature of the solvent, and the chemical nature of the middle block on the morphology and topography, Figure 5 shows the AFM images obtained for various copolymers under different conditions.

In order to analyze the effect of solution concentration, morphologies obtained for the SIS copolymer casted from toluene solutions of 1 and 3 wt%, annealed at RT, are shown as an example (Figure 5a,b). Films prepared from 1 wt% solutions showed a mixture of perpendicular and parallel cylinders, while, for those prepared from 3 wt% solutions, only parallel cylinders were observed. Furthermore, smaller cylinders could be observed at 1 wt% (18.1 nm vs. 25.1 nm), probably because the outermost surface was closer to the substrate, affecting the final morphology. The sample’s surface at 3 wt% was smoother than at 1 wt% (5.65 vs. 7.84).

In order to analyze the effect of solvent nature, morphologies for SIS prepared from 3 wt% solutions in toluene, cyclohexane, and THF, annealed at RT, are presented in Figure 5b–d, respectively. The preferential affinity of the solvent can increase the volume of a specific block. Solvent molecules are present in this block for longer times; thus, relaxation and stretch configurations can be easily reached [47,48]. As solvent evaporates from the blocks, junction points at the interfaces move closer in order to minimize the interfacial area, leading to an enhancement of the domain size [49]. At RT, PS blocks are below their glass transition temperature (T_g_), and, for the case of cyclohexane, the outermost surface is mainly PI (also due to the surface energy of PI being lower than that of PS). The affinity of toluene is higher for PI, while that of THF is higher for PS. Therefore, a mixture of blocks in the outermost surface can be expected. The much slower evaporation rate of toluene creates almost 100% parallel cylinders (Figure 5b), while, for THF, the fast evaporation led to a mixture of perpendicular and parallel ones. The case of cyclohexane was somewhere in the middle, with a higher probability of PI being on the outermost surface, but with an intermediate evaporation rate. Thus, a morphology based on parallel cylinders with perpendicular ones in some areas was found. For similar systems (SBS with 30 wt% PS) a connection between evaporation rate and orientation of cylinders was observed, where the fastest evaporation rate resulted in a disordered structure, while a slower rate resulted in self-assembled nanostructures with perpendicular, perpendicular/parallel, and parallel cylinders [46]. Fast solvent evaporation allows cylinders to grow in the direction of maximum solvent concentration, adopting a perpendicular orientation, while slower evaporation presents less kinetic constraints for parallel cylinders [46]. Regarding roughness, the highest value was found for THF, followed by cyclohexane and toluene, due to their polarity and evaporation rate (11.38, 6.05, and 5.65, respectively).

The effect of the copolymer nature was analyzed by comparing SIS, SEPS, and SBS samples casted from 3 wt% solutions in toluene, annealed at RT (Figure 5b,e,f, respectively). SIS presented a morphology of parallel cylinders, while a worm-like morphology with perpendicular cylinders was obtained for SEPS, and perpendicular disordered cylinders were obtained for SBS [46]. The differences were mainly due to the way in which the middle block orientated toward the surface, to its immiscibility with PS, and its affinity for toluene. The different affinity of the middle block for toluene with respect to that of PS played the most important role, as seen in Table 2 (0.170 for PS, 0.036 for PI, 0.091 for PB, 0.120 for PEP, and 0.039 for the PE), in the generation of those nanostructures. The highest affinity of PI for toluene resulted in the solvent having more contact with the PI chain, together with a higher aggregation of PS, leading to a highly organized structure of parallel cylinders. In the case of PB, perpendicular cylinders could be seen, albeit disordered. PEP showed a worm-like morphology with perpendicular cylinders, mainly due to the similar affinities of PS and PEP. These results could be also verified from the roughness values: 8.67 for SEPS, 5.65 for the parallel cylinders of SIS, and 3.31 for the perpendicular cylinders of SBS, due to the morphology and the nature of the middle block.

The final part of the analysis involved the effect of solvent and annealing temperature on the size of nanostructures. A solvent with higher affinity for one of the blocks offers it an extra volume, allowing an increase in domain size, as junction points get closer in order to minimize the interfacial area [49]. Comparing the SEPS/SIS and SEES/SBS systems, some conclusions on the size of the cylinders and worm-like morphology, as well as on the shape and size of crystals could be extracted. The SBS copolymer casted from cyclohexane presented sizes of around 27 nm for PS, and of around 17 nm for PB, increasing to 22 nm for toluene as it became more swollen. For the case of SEES, the worm-like morphology showed similar sizes (around 29 nm for PS, and slightly higher for PEE than PB, at around 25 nm), due to the higher affinity of PEE compared to PB for cyclohexane and toluene.

The SIS and SEPS system was more complicated to analyze since it resulted in more organized nanostructures and crystal-like structures. In the case of SEPS, it is interesting to refer to the worm-like morphology and the change in sizes. At 1 wt% concentration, the PS domain sizes were around 28 nm (cyclohexane), 38 nm (THF), and 32 nm (toluene), in agreement with the affinity for the solvents. Similarly, sizes of 22 nm (cyclohexane), 23 nm (THF), and 36 nm (toluene, with better affinity for PEP chains) were obtained for PEP. At 3 wt% concentration, the results were similar those mentioned above for the worm-like morphology. For more organized morphologies and with an increase in temperature (1 wt%, cyclohexane and toluene), sizes seem to be bigger, around 22 nm for PS at RT, increasing to 30 nm at 60 °C, while, for PEP, a size of around 10 nm was found at RT, increasing to 28 nm at 60 °C, all in cyclohexane. Similarly, for the case of toluene, sizes were bigger up to 60 °C; however, at 80 °C, they become smaller, probably due to the nucleation of PEP crystals of around 104 nm at 60 °C and 124 nm at 80 °C. Also, compared to crystals obtained in cyclohexane (64 nm at 60 °C), it is clear that the affinity of PEP for toluene was a crucial factor for the enhanced size of the crystals. For the case of cyclohexane with 3 wt%, the size of PEP domains decreased with temperature (20 to 19 to 17 to 12 nm), while that of PS domains showed an irregularity (25 to 23 to 24 to 29 nm), obtaining the highest size after annealing at 100 °C.

Finally, for SIS, it should be noted that the worm-like morphology for samples of toluene with 1 wt% produced sizes of 28 nm 22 nm for PS and PI, respectively. It seems that a polydispersity of sizes existed and it was affected by temperature, solution concentration, and solvent. Both PS and PI showed a better affinity for toluene than cyclohexane, resulting in large crystal-like domains for both polymers (PS from 24 nm to 27 nm, and PI from 15 nm to 18 nm), as obtained in films casted from 1 wt% solutions of both solvents, annealed at RT.

## 4. Conclusions

From the results, useful conclusions concerning the effect of solvent nature, solution concentration, and annealing temperature on the ordering of ABA-type SIS and SBS copolymers and their hydrogenated SEPS and SEES analogs could be derived.

Worm-like and cylindrical morphologies were the most common morphologies observed with some differences, such as the appearance of crystals, a mixture of parallel and perpendicular cylinders, and a worm-like morphology with cylinders. Depending on the nature of the block and the solvent, a different degree of ordering and orientation could be observed. Regarding the annealing temperature, at RT, almost all types of morphologies were observed. For intermediate annealing temperatures (60 and 80 °C), intermediate structures and the appearance of crystal-like domains were observed, while, for samples annealed at 100 °C, disordered and worm-like nanostructures were observed.

The affinity of the solvent for each block, in combination with the evaporation rate, was a key factor for the final obtained nanostructure. THF had an affinity for PS, while toluene/cyclohexane had an affinity for polydiene, leading to the formation of worm-like morphologies (THF) and cylindrical ones (toluene/cyclohexane). The most ordered nanostructures were observed for SEPS, indicating that immiscibility among blocks was also an important parameter.

The affinity of the solvent for each block offered an extra volume to that block, while the remainder tended to segregate in order to avoid the solvent. Therefore, blocks were organized onto the glass substrate in different ways depending on the solvent and temperature.

In conclusion, the ordering of block copolymers into different nanostructures is a multi-faceted procedure, in which, in addition to the nature of the block, other parameters of film preparation play a significant role: (i) solvent (polarity and evaporation rate), (ii) temperature (thermodynamic equilibrium), and (iii) polymer concentration. All these parameters can be varied to obtain desired ordered nanostructures with different orientations and degrees of ordering.

## Figures and Tables

**Table 1:** Characteristic dispersion solubility (δ_d_), dipole solubility (δ_p_) and hydrogen-bond solubility (δ_h_) parameters, for all solvents and polymeric blocks.

Dispersion Parameters	δ_d_ (MPa^1/2^) ^a^	δ_p_ (MPa^1/2^) ^a^	δ_h_ (MPa^1/2^) ^a^	V (cm^3^/mol) ^b^
**Solvents**				
Toluene	18.0	1.4	2.0	106.8
Cyclohexane	16.8	0.0	0.2	108.7
Tetrahydrofuran (THF)	16.8	5.7	8.0	81.7
**Polymeric blocks**				
Polystyrene (PS)	17.6	6.1	4.1	-
Polybutadiene (PB)	18.0	5.1	2.5	-
Poly(isoprene) (PI)	17.4	3.1	3.1	-
Poly(ethylene/propylene) (PEP)	16.7	0.0	5.2	-
Poly(ethylene) (PE)	18.1	0.0	0.0	-

^a,b^ All solubility parameters, as long as solvent volume were found in the literature, were calculated according to Hansen solubility parameters [51] and the van Krevelen method [64].

**Table 2:** Characteristic results for the Flory–Huggins χ_sp_ parameter between used solvents and polymeric blocks, together with the affinity of each block for all solvents.

Polymers	Toluene	Cyclohexane	THF	Affinity
PS	0.170	0.360	0.088	THF > Toluene> Cyclohexane
PB	0.091	0.241	0.178	Toluene > THF > Cyclohexane
PI	0.036	0.127	0.160	Toluene > Cyclohexane > THF
PEP	0.120	0.160	0.200	Toluene > Cyclohexane > THF
PE	0.039	0.044	0.506	Toluene > Cyclohexane > THF

All χ_sp_ values were calculated using the equation, χ_sp_ = (aV/RT) × ((δ_ds_ − δ_dp_)^2^ + 0.25(δ_ps_ − δ_pp_)^2^ + 0.25(δ_hs_ − δ_hp_)^2^), where V (cm^3^/mol) values were taken from Table 1, R = 8.314 cm^3^MPa/K·mol, and *a* is a constant with a value of 0.6 [52]; δ_d_, δ_p_, and δ_h_ (MPa) values were taken from Table 1.

**Table 3:** Characteristic morphologies observed for all samples with different solvents (cyclohexane, toluene, and THF), annealing temperatures (room temperature (RT), and 60, 80 and 100 °C), and concentrations (1 and 3 wt%). SEPS—poly(styrene-b-ethylene)/poly(propylene-b-styrene); SIS—poly(styrene-b-isoprene-b-styrene); SEES—poly(styrene-b-ethylene)/poly(ethylene-b-styrene); SBS—poly(styrene-b-butadiene-b-styrene).

Morphologies	SEPS		SIS		SEES		SBS	
	1%	3%	1%	3%	1%	3%	1%	3%
Disordered	-	-	4C,3C 4T,3T 2F,3F 4F	4C,3C 4T,3T 3F	1C,2C 3C,1T 2T,3T 1F,2F 3F	1C,2C 3C,1T 2T,3T 4T,1F 2F	2C,3C 4C,2T 4T,1F 2F,3F 4F	2C,3C 4C,3T 4T,1F 2F,3F 4F
Worm-like	4C,3C 4T,1F 2F,3F 4F	4T,1F 2F,3F 4F	2T,1F	-	4C,4T 4F	4C,4F 3F	1C,3T	-
Worm-like + crystals	2C,3T 2T	-	-	-	-	-	-	-
Worm-like + perpendicular cylinders	-	1C,2C 3C,4C 1T,2T	-	-	-	-	-	2T
Parallel cylinders	-	-	1C	1C,1T	-	-	-	1C
Perpendicular cylinders + crystal-like domains	-	-	2C	2C,2T 4F,2F	-	-	-	-
Perpendicular cylinders (disordered)	-	-	-	-	-	-	1T	1T
Perpendicular + parallel cylinders	1C,1T	3T	1T	1F	-	-	-	-

Table is divided for the four different samples on the basis of concentration (1 wt%) and (3 wt%). The abbreviations for each sample are referred to in an XY format, where X denotes the temperature and Y denotes the solvent. All parameters (concentration, solvent type, and temperature) are listed in Table 4.

**Table 4:** Different parameters used for all samples with appropriate abbreviations, as used in Table 3.

Parameters				
Concentration	1%	3%	-	-
Solvent	Toluene (T)	THF (F)	Cyclohexane (C)	-
Temperature	RT (1)	60 °C (2)	80 °C (3)	100 °C (4)

## Supplementary Materials

The following are available online at http://www.mdpi.com/1996-1944/11/9/1529/s1: an analysis of film roughness, and Figure S1: evolution of surface average RA roughness with temperature for SBS, SEES, SIS, and SEPS films prepared with (a) different solvents and (b) solution concentrations.
