# Supplementary material for: Exploring the Self-Assembly Capabilities of ABA-Type SBS, SIS, and Their Analogous Hydrogenated Copolymers onto Different Nanostructures Using Atomic Force Microscopy
Source: Materials (Basel). 2018 Aug 24;11(9):1529. doi: 10.3390/ma11091529 (PMC6165061; doi:10.3390/ma11091529)
Supplement: Supplementary file 1 [file materials-11-01529-s001.pdf]

*materials*

MDPI

*Supporting Information*

# Exploring the Self-assembly Capabilities of ABA-Type SBS, SIS, and Their Analogous Hydrogenated Copolymers onto Different Nanostructures Using Atomic Force Microscopy

**Nikolaos Politakos [1,2]* and Galder Kortaberria [2]**

[1] POLYMAT and Departamento de Química Aplicada, Facultad de Ciencias Químicas, University of the Basque Country UPV/EHU, Joxe Mari Korta Center, Avda. Tolosa 72, 20018 Donostia - San Sebastian, Spain; nikolaos.politakos@ehu.eus

[2] "Materials + Technologies" Group, Chemical & Environmental Engineering Department, Basque Country University, Plaza Europa 1, 20018 Donostia, Spain; galder.cortaberria@ehu.eus

* Correspondence: Nikolaos Politakos, POLYMAT and Departamento de Química Aplicada, Facultad de Ciencias Químicas, University of the Basque Country UPV/EHU, Joxe Mari Korta Center, Avda. Tolosa 72, 20018 Donostia - San Sebastian, Spain nikolaos.politakos@ehu.eus; Tel.: +34-943018475

## Film Roughness Analysis

An evaluation of film surface roughness was carried out using AFM for all samples. The average roughness ($R_A$) of 1 × 1 μm images was calculated. In Figure S1, the evolution of $R_A$ is shown for all polymeric films prepared with different solvents at different concentrations.

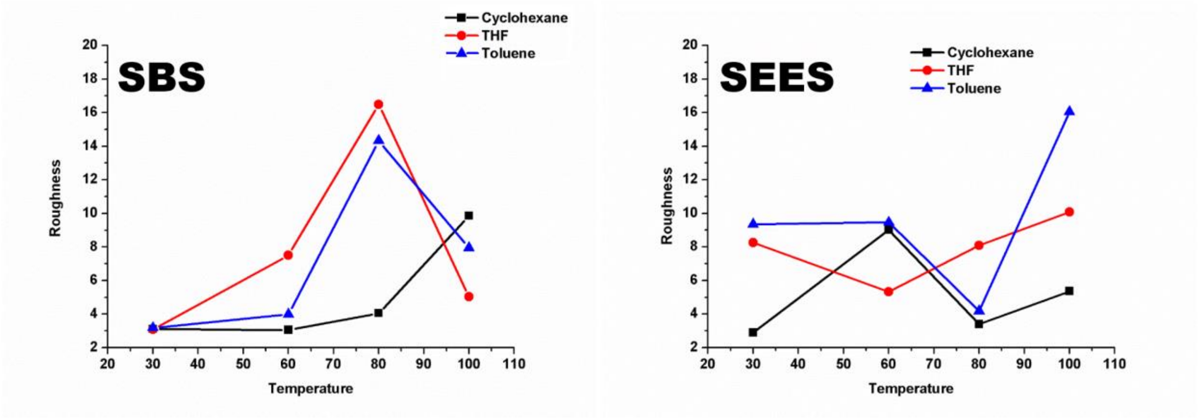

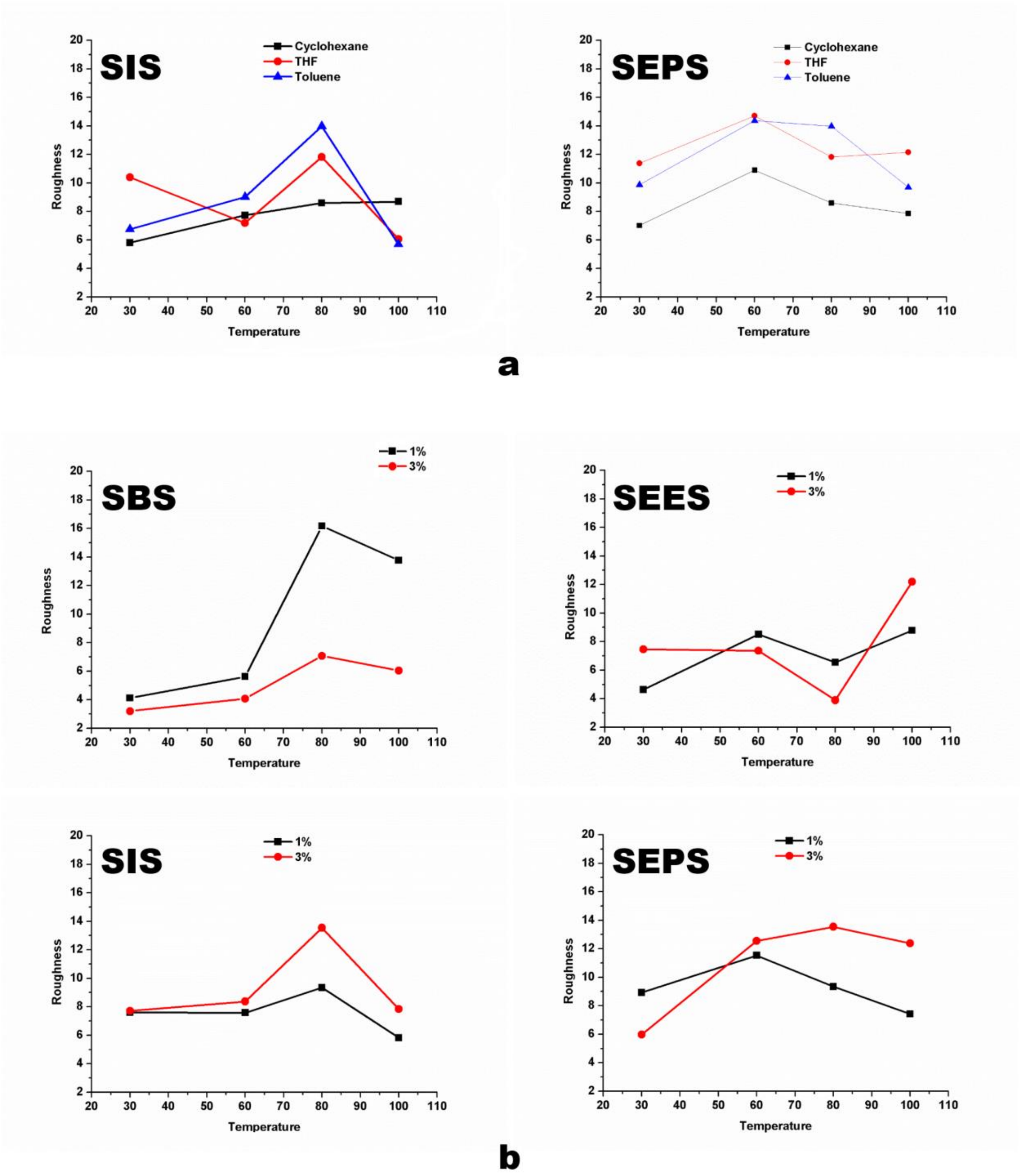


**Figure S1.** Evolution of average surface roughness ($R_A$) with temperature for SBS, SEES, SIS, and SEPS films prepared with (**a**) different solvents and (**b**) solution concentrations.

The hydrogenation of middle blocks resulted in PE/PP and PE in addition to initial PI and PB, with the change in the middle block leading to rougher surfaces, related to the stronger repulsion among blocks. PE/PP and PE presented higher immiscibility than PS chains with PB and PI, resulting in rougher surfaces than their precursors. Among SEPS and SEES, the PP/PE middle block of the former resulted in rougher surfaces when compared to PE.

Film roughness was also affected by annealing temperatures and employed solvents. Polymeric systems of SBS and SIS showed the same tendency in roughness concerning the polymer concentration, whereby roughness increased until 80 °C before dropping. A more mixed tendency was observed for SEES and SEPS, where the presence of crystals or the dominant worm-like morphology affected the roughness for intermediate temperatures, at which point the morphologies formed. For the case of different solvents, a mixed behavior can be seen, since the evaporation rate of the solvent was important for the formation of the polymeric film, and subsequently, for the morphology. For SBS, a tendency toward higher roughness was found for the intermediate temperatures, which continued in the case of the cyclohexane, whereas it dropped for the other two solvents. The analogous SEES had a mixed roughness going from RT to intermediate temperatures (increasing and decreasing); however, at 100 °C, the roughness increased for all solvents. SIS was similar to SBS, where, at intermediate temperatures, roughness increased and continued for the case of cyclohexane. SEPS showed a decrease in roughness for intermediate temperatures, which decreased further at 100 °C for cyclohexane and toluene (where enough time was given due to the evaporation rate), in addition to THF. SEPS was also the polymeric system with the most organized nanostructures at many temperatures, creating smoother surfaces.

Upon increasing the annealing temperature, the presence of crystalline or crystal-like domains (as shown in Figures 1 and 2) led to higher values of $R_A$. Finally, another explanation for the lower roughness at the higher annealing temperature of 100 °C was the disappearance of domains present at 60 and 80 °C, as seen in the AFM images.

Regarding the employed solvent, in addition to its effect on the evaporation rate, chains may or may not have enough time to relax and adopt configurations, with a clear effect on roughness also observed. Taking into account that the solvent polarity is directly related to the affinity for each block, [1–6] an increase in solvent polarity led to higher roughness values (as clearly seen, especially for SBS and SEPS). This fact could also be related to the evaporation rate, which was very high for THF, with polymeric chains having less time to relax and order. The evaporation rate/affinity combination was the main factor giving rise to the final roughness of each sample. Cyclohexane presented a higher tendency to separate the middle block from PS (as it is a Θ solvent for PS at RT, whereby affinity increases with temperature [38]). In addition, toluene presented a higher affinity for the middle block and the slowest evaporation rate; thus, blocks had time for relaxation and ordering, leading to smoother surfaces.

## References

1. Kim, G.; Libera, M. Morphological Development in Solvent-Cast Polystyrene−Polybutadiene−Polystyrene (SBS) Triblock Copolymer Thin Films. Macromolecules 1998, 31, 2569-2577, DOI: 10.1021/ma971349i.
2. a) Guo, R.; Huang, H.; Du, B.; He, T. Solvent-Induced Morphology of the Binary Mixture of Diblock Copolymer in Thin Film: The Block Length and Composition Dependence of Morphology. Journal of Physical Chemistry B 2009, 113, 2712-2724, DOI: 10.1021/jp808551j. b) Guo, R.; Huang, H.; Chen, Y.; Gong, Y.; Du, B.; He, T. Effect of the Nature of Annealing Solvent on the Morphology of Diblock Copolymer Blend Thin Films. Macromolecules 2008, 41, 890-900, DOI: 10.1021/ma701979q.
3. Gong, Y.; Huang, H.; Hu, Z.; Chen, Y.; Chen, D.; Wang, Z.; He, T. Inverted to Normal Phase Transition in Solution-Cast Polystyrene−Poly(methyl methacrylate) Block Copolymer Thin Films. Macromolecules 2006, 39, 3369-3376, DOI: 10.1021/ma052380k.

4. Huang, H.; Zhang, F.; Hu, Z.; Du, B.; He, T.; Kay Lee, F.; Wang, Y.; Tsui, O. K. C. Study on the Origin of Inverted Phase in Drying Solution-Cast Block Copolymer Films. Macromolecules 2003, 36, 4084-4092, DOI: 10.1021/ma0217581.
5. Zhang, Q.; Tsui, O. K. C.; Du, B.; Zhang, F.; Tang, T.; He, T. Observation of Inverted Phases in Poly(styrene-b-butadiene-b-styrene) Triblock Copolymer by Solvent-Induced Order−Disorder Phase Transition. Macromolecules 2000, 33, 9561-9567, DOI: 10.1021/ma001161q.
6. Huang, H.; Hu, Z.; Chen, Y.; Zhang, F.; Gong, Y.; He, T. Effects of Casting Solvents on the Formation of Inverted Phase in Block Copolymer Thin Films. Macromolecules 2004, 37, 6523-6530, DOI: 10.1021/ma0498621.
